# The emergence of DNAM-1 as the facilitator of NK cell-mediated killing in ovarian cancer

**DOI:** 10.3389/fimmu.2024.1477781

**Published:** 2025-01-06

**Authors:** Rachel Pounds, Wayne Croft, Hayden Pearce, Tasnia Hossain, Kavita Singh, Janos Balega, David N. Jeevan, Sudha Sundar, Sean Kehoe, Jason Yap, Paul Moss, Jianmin Zuo

**Affiliations:** ^1^ Institute of Cancer and Genomic Sciences, College of Medical and Dental Sciences, University of Birmingham, Birmingham, United Kingdom; ^2^ Pan-Birmingham Gynaecological Cancer Centre, City Hospital, Birmingham, United Kingdom; ^3^ Institute of Immunology and Immunotherapy, College of Medical and Dental Sciences, University of Birmingham, Birmingham, United Kingdom; ^4^ Oxford Gynaecological Cancer Centre, Churchill Hospital, Oxford University Hospitals Foundation Trust, Oxford, United Kingdom

**Keywords:** HGSOC, NK cells, DNAM-1, cancer immunology, ovarian cancer

## Abstract

**Introduction:**

Ovarian cancer (OC) is the sixth most common malignancy in women and the poor 5-year survival emphasises the need for novel therapies. NK cells play an important role in the control of malignant disease but the nature of tumour-infiltrating and peripheral NK cells in OC remains unclear.

**Methods:**

Using flow cytometric analysis, we studied the phenotype and function of NK cells in blood, primary tumour and metastatic tissue in 80 women with OC. The cell type contexture of metastatic OC tissue was explored utilising scRNAseq analysis, with a focus on portraying an immunogenic tumour microenvironment and determining the characteristics of a dysfunctional NK cell population.

**Results:**

The proportion of peripheral NK cells was markedly elevated with a highly activated profile and increased cytotoxicity. In contrast, NK cell numbers in primary tumour and metastasis were substantially reduced, with downregulation of activatory receptors together with elevated PD-1 expression. scRNA-Seq identified 5 NK cell subpopulations along with increased exhausted and immature NK cells within tumour tissue compared to normal tissue. These features were attenuated following chemotherapy where higher levels of activated and cytotoxic NK cells associated with improved disease-free survival. Correlation of NK cell phenotype with clinical outcomes revealed high levels of DNAM-1 expression on tissue-localised and peripheral NK cells to be associated with reduced survival. Expression of PVR, the DNAM-1 ligand, was significantly increased on tumours and DNAM-1 mediated NK cell lysis of primary tumour tissue was observed *in vitro*.

**Discussion:**

These findings reveal profound modulation of the tumour tissue and systemic profile of NK cells which likely contributes to the high rates of local progression and metastasis seen with OC. Immunotherapeutic approaches that overcome local immune suppression and enhance DNAM-1-targeted lysis of OC offer the potential to improve disease control.

## 1 Introduction

Ovarian cancer (OC) is the sixth most common cancer amongst women, with approximately 314,000 new cases diagnosed globally each year (1). Around 90% are of epithelial cell (EOC) origin, with the most common and aggressive subtype being high-grade serous ovarian cancer (HGSOC) (2–5). The majority of patients have advanced disease (FIGO stage III/IV) at diagnosis, where tumours have spread beyond the pelvis (2). Current standard treatment consists of platinum-based chemotherapy and cytoreductive surgery (6). Surgical intervention aims to remove all macroscopically visible disease which is associated with improved survival outcomes (7). Nevertheless, only one third are alive and disease free 18 months after completion of treatment, with 5-year survival rates of 15% and 25% for stages III and IV, respectively (2, 8). As such, there is an urgent need to identify novel therapeutic approaches for these women (2, 8, 9).

The presence of neoplastic cells can elicit a tumour-specific immune response, yet the tumour microenvironment (TME) can also act to suppress the immune infiltrate and support tumour survival and progression (10–12). The nature of the immune infiltrate within the ovarian TME has an important impact on prognosis, with HGSOC demonstrating the highest level of intra-tumoral infiltration (12, 13). In addition, increased numbers of tumour-infiltrating lymphocytes (TILs), particularly CD8+ cells, are associated with longer survival (14, 15). Checkpoint blockade has been a successful immunotherapy approach for many cancer subtypes but meta-analysis has established that PD-1/PD-L1 inhibitors have limited clinical efficacy in HGSOC (16).

Natural Killer (NK) cells are a critical component of the innate immune system and have the ability to destroy neoplastic cells in an antigen-independent manner (17–20). However, tumour-infiltrating NK cells display low levels of proliferation and activation, and in some malignancies display a regulatory phenotype (21–23). NK cells have prognostic implications for OC patients with improved clinical outcomes associated with a greater total NK cell number, increased peripheral blood NK cells, high levels of NK activation, and increased penetration into ovarian tumour tissues (24, 25).

NK cells use a range of different methods to target neoplastic cells and overcome tumour escape mechanisms, and as such there is the potential for application of NK immunotherapies in OC (11, 17). However, the achievement of this will require further understanding of NK cell biology and its relation to clinical outcomes. Here we report a detailed characterisation of the NK cell transcriptome, phenotype and function in OC patients who underwent treatment with curative intend. Our findings show the significance of DNAM-mediated lysis of ovarian tumour cells and could help to guide the introduction of stratified immunotherapy protocols in women with OC.

## 2 Materials and methods

### 2.1 Patient cohort

A total of 80 patients with OC were recruited, of which 40 underwent primary surgery followed by 6 cycles of platinum-based adjuvant chemotherapy. The remaining 40 patients received neoadjuvant chemotherapy (NACT) followed by interval debulking surgery and additional adjuvant chemotherapy. A further 20 patients were recruited as healthy donor (HD) controls: 10 patients with a benign ovarian mass and 10 with histologically normal ovaries and fallopian tubes. All patients were age-matched (demographics shown in **Table 1**) and were recruited using approved ethics (IRAS ID 225991). Peripheral blood samples were taken from all patients immediately prior to anaesthetic induction in BD Vacutainer heparin tubes. Tissue biopsies were obtained from ovarian and omental tissues at the time of surgical resection and were stored at 4℃ in basic culture media (sterile RPMI 1640 media (Fisher Scientific Ltd; 15450564) with 2mM L-glutamine (Thermo Fisher Scientific (RRID: SCR_008452); 25030081), 10% sterile heat-inactivated foetal bovine serum (FBS; Thermo Fisher Scientific (RRID: SCR_008452); 11550356) and 1% sterile antibiotics (penicillin 10,000 units/ml and streptomycin 10mg/ml; Sigma-Aldrich (RRID: SCR_008988); P4333)).

**Table 1:** Patient characteristics, histopathological findings and treatment variables.

Variable	Normal ovary (n=10)	Benign ovary (n=10)	Primary surgery (n=40)	Neoadjuvant chemotherapy (n=40)	P value
**Age (years)** Mean ± SD	57.3 ± 13.7	62.1 ± 12.8	60.7 ± 14.6	64.5 ± 11.6	0.4480
**Ethnicity** White Asian Black Mixed	**n (%)** 7 (70.0) 1 (10.0) 1 (10.0) 1 (10.0)	**n (%)** 6 (60.0) 2 (20.0) 1 (10.0) 1 (10.0)	**n (%)** 33 (82.5) 5 (12.5) 0 (0.0) 2 (5.0)	**n (%)** 32 (80.0) 6 (15.0) 1 (2.5) 1 (2.5)	0.2960
**FIGO stage** 1 2 3 4	n/a	n/a	**n (%)** 11 (27.5) 1 (2.5) 21 (52.5) 7 (17.5)	**n (%)** 0 (0.0) 0 (0.0) 21 (52.5) 19 (47.5)	0.7429
**PCI** Median ± IQR	n/a	n/a	11 (3-15)	16 (5-18)	0.1485
**Histopathology** Serous Clear cell Mucinous Endometrioid	n/a	n/a	**n (%)** 34 (85.0) 3 (7.5) 2 (5.0) 1 (2.5)	**n (%)** 39 (97.5) 0 (0.0) 0 (0.0.) 1 (2.5)	0.4000
**Cytoreduction** ≤ 1cm >1cm n/a	n/a	n/a	**n (%)** 34 (85.0) 4 (10.0) 2 (5.0)	**n (%)** 27 (67.5) 1 (2.5) 12 (30.0)	0.1274
**Number NACT cycles** 3 4 5 6	n/a	n/a	n/a	**n (%)** 15 (37.5) 12 (30.0) 3 (7.5) 10 (25.0)	n/a
**Chemotherapy Response Score** 1 2 3 n/a	n/a	n/a	n/a	**n (%)** 17 (42.5) 14 (35.0) 8 (20.0) 1 (2.5)	n/a

PCI, peritoneal cancer index; Cytoreduction, residual disease ≤ 1cm or >1cm.

Chemotherapy Response Score 1: poor response, 2: moderate response, 3: complete response.

### 2.2 Flow cytometry analysis of NK cells

Peripheral blood mononuclear cells (PBMCs) were isolated using density gradient centrifugation. Tissue samples were sectioned into 2mm diameter pieces, digested with Collagenase (Life Technologies # 17101015, 2.5mg/ml) and single cells were collected by centrifugation and resuspended in PBS. A total of 1x10^6^ cells were transferred into FACS tubes for each antibody panel. The NK cell activating panel included: CD3 - FITC (Biolegend 344804), CD16 - PE (Biolegend 302008), CD14 - ECD (Beckman Coulter B92391), CD19 - ECD (Beckman Coulter 6604551), PI - ECD (Miltenyi Biotec 130093233), NKp46 - PerCP-Cy5.5 (Biolegend 137610), CD56 - PE/Cy7 (Biolegend 318318), NKG2C - APC (Biolegend 250704), CD45 - AF700 (Biolegend 304024), DNAM-1 - APC-Fire750 (Biolegend 338320), CD57 - PB (Biolegend 359608) and NKG2D - BV510 (Biolegend 320816). The NK inhibitory panel included: CD3 - FITC (Biolegend 344804), PD-1 - PE (Biolegend 329905), CD14 - ECD (Beckman Coulter B92391), CD19 - ECD (Beckman Coulter 6604551), PI - ECD (Miltenyi Biotec 130093233), CTLA-4 - PerCP-Cy5.5 (Biolegend 349928), CD56 - PE/Cy7 (Biolegend 318318), TIGIT - APC (Biolegend 372706), CD45 - AF700 (Biolegend 304024), CD94 - APC-Fire750 (Biolegend 305518), CD16 - PB (Biolegend 302008) and CD69 - BV510 (Biolegend 310936). Following incubation on ice for 30 minutes, samples were processed on the Gallios flow cytometer (Beckman Coulter Life Sciences). Kaluza, Flow Cytometry Analysis Software (Beckman Coulter, analysis version 2.1) was used to analyse flow cytometry data.

### 2.3 NK cytotoxicity analysis

NK cell enrichment was performed using The EasySep™ Human NK cell Isolation kit (STEMCELL Technologies 17955). Patient PBMC samples were processed to generate an isolated NK cell suspension of 1x10^6^ cells in 1ml basic culture media. Authenticated target cell K-562 (RRID: CVCL_0004; sourced from ATCC (26)) was labelled with Carboxyfluorescein succinimidyl ester (CFSE; eBioscience; 65-0850-84) and co-cultured with NK cells in a 1:5 ratio in 96 well plates. Following 16-hour incubation at 37℃, cells were harvested and 10µl CountBright™ beads (Thermo Fisher Scientific (RRID: SCR_008452); C36995) added to generate absolute K-562 cell counts. The Gallios flow cytometer and Kaluza Analysis Software determined the viable K-562 cell concentration, and subsequently the degree of NK killing.

To assess NK cytotoxicity towards ovarian tissue, processed ovarian specimens were co-cultured in a 1:1 ratio with NK cells obtained from both autologous and healthy donor PBMC samples. Following overnight incubation at 37℃, cells were stained with EPCAM - BV421 (Biolegend 32420), HLA-1 – PE/Cy7 (Biolegend 311430) and CD56 - AF700 (Biolegend 318316). Samples were analysed on the Gallios flow cytometer and Kaluza Analysis Software determined live cell counts and the degree of killing by NK cells. Ovarian - NK cell co-cultures were also generated with blocking antibodies: anti-DNAM-1 (Biolegend 338302), anti-NKG2D (Biolegend 320813), anti-NKp46 (Biolegend 331947) and anti-NKp30 (Biolegend 325223).

### 2.4 NK cytokine assay

Processed PBMC samples (1x10^6^ cells) were cultured alone, in combination with K-562 cells (1:1 ratio) and with the addition of eBioscience™ cell stimulation cocktail (500X; Thermo Fisher Scientific (RRID: SCR_008452); 00-4970-93). Following overnight incubation at 37℃, cells were stained with viability dye (LIVE/DEAD™ fixable red dead cell stain for 488nm excitation; Thermo Fisher Scientific (RRID: SCR_008452); L23102) and surface antibodies: CD14 - ECD (Beckman Coulter; B92391), CD19 - ECD (Beckman Coulter; 6604551), viability dye - ECD (Thermo Fisher Scientific (RRID: SCR_008452); 15530795) CD3 - PerCP/Cy5.5 (Biolegend; 300327), TGF Beta - APC (Biolegend;141405), CD56 - APC-Cy7 (Biolegend 318331) and CD16 - BV421 (Biolegend 302037) before they were fixed and permeabilized. Cells were then stained with intracellular antibodies: TNF alpha - FITC (Biolegend 502906), GM-CSF – PE (Biolegend 502305), IL-10 – PE-Cy7 (Biolegend 501419), IFN gamma - AF700 (Biolegend 506515) and IL-2 – BV510 (Biolegend 503833), and were processed by the Gallios flow cytometer before examination using Kaluza Analysis Software. The gating strategy implemented to identify NK cell populations within each blood and tissue sample following flow cytometry is shown in **Supplementary Figure 1**.

### 2.5 Real-time quantitative PCR of NK cell ligands

Ovarian biopsies from benign and malignant specimens were snap frozen immediately after surgical resection and preserved in vapor phase nitrogen storage. RNA extraction was undertaken using the RNeasy Mini Kit (Qiagen; 74104) and iScript cDNA Synthesis Kit (BioRAD; 1708890) converted RNA into complementary DNA (cDNA). QRT-PCR assays for PVR (Hs00197846_m1) and PVRL2 (Hs00607609_m1) were performed with TaqMan Gene Expression Assays (Applied Biosystems), with 18s assayed for normalization. For absolute quantification, reference plasmids containing relevant PCR target gene sequences were used to generate standard curves. The data was evaluated using SDS software version 1.5 (Applied Biosystems).

### 2.6 Tissue processing for single cell RNA sequencing

Omental samples were utilised for scRNAseq analysis from 9 women with stage III/IV HGSOC. The omentum was sectioned into 2mm size pieces immediately following surgical resection and digested using collagenase (1500 units), hyaluronidase (500 units; StemCell Technologies; 07912), liberase TL (500µl; Sigma-Aldrich (RRID: SCR_008988); 05401020001) and benzonase nuclease (500 units; Merck; E1014) at 37°C for 90 minutes. The resulting single cell solution was centrifuged, added to 2ml red blood cell lysis buffer (Cambridge, Bioscience; catalogue number RBCL-1) and washed with 4ml sterile MACS buffer (PBS, 2mM of EDTA (Corning) and 2% of newborn calf bovine serum (Thermo Fisher Scientific (RRID: SCR_008452)). Samples were stained with 15µl Fc receptor blocking solution (Biolegend; 422302) and antibodies CD45 - BV785 (Biolegend 304047), Epcam - APC (Biolegend 324207) and Podoplanin - FITC (Biolegend 337025), before being suspended in 500µl MACS buffer for cell sorting. Here cells were separated into immune cells (high CD45 expression), epithelial cells (high Epcam and low podoplanin expression), and fibroblasts (low Epcam and high podoplanin expression). Cell populations were combined, with CD45 positive cells making up 75% and the Epcam/podoplanin cells 25% of the final sample, in a concentration of 1 x 10^6^ cells per ml of basic media. This reconstruction of single-cell samples ensured a detailed immunogenomic analysis was conducted and also allowed adequate epithelial tumour cell evaluation.

### 2.7 Library preparation for single cell RNA sequencing

The samples were processed with the 10X Genomics Chromium Controller and the Single Cell 3’ GEM, Library and Gel Bead kit (version 3.1; 10X Genomics; catalogue number PN-1000121) (27). Gel beads-in-emulsion (GEMs) were produced, within which the individual cells were lysed, the mRNA tagged with a 10X barcode and unique molecular identifier (UMI), and reverse transcription performed to produce cDNA. The cDNA was amplified using PCR before being fragmented and primer sequences added via end repair, A-tailing and adaptor ligation. The cDNA was then size selected with Solid Phase Reversible Immobilization (SPRI)-select reagents, cleaned using magnetic beads and the library amplified with PCR. Illumina NEXTSeq was then used to sequence the sample, identifying 20,000-40,000 reads per cell.

### 2.8 Analysis of the single cell RNA sequencing data

The raw sequencing reads were processed using Cell Ranger (version 5.0.1) (28, 29). Reads were aligned to the Human Reference Genome (version GRCh38) with cell ranger count and raw UMI (unique molecular identifier) matrices were further analysed using “R” (version 3.6.2). Any doublets within the dataset (identified with DoubletFinder (RRID: SCR_018771) version 3.0 (30) were removed. In addition, cells were eliminated if they had fewer than 500 genes detected, more than 3500 genes detected or if a high proportion (> 10%) of their genes mapped to mitochondrial RNA. Genes were filtered to remove those expressed in 3 or less cells. Downstream analysis was conducted with Seurat (version 3.2.0 (31). Cell cycle scores were calculated using Seurat CellCycleScoring function and the difference between G2M and S phase score quantified. Seurat SCTransform pipeline was followed for normalisation, regressing out % mitochondrial mapping and G2M-S phase score difference. SCTransform-normalised data for each sample was integrated following the SCTransform integration workflow on the top 8000 most variable features. The most variable features were used for dimensionality reduction by principal component analysis and subsequent uniform manifold projection with PCs 1:20 that explained most of the variance.

### 2.9 Unsupervised clustering and major cell lineage annotation

To identify major lineage cell types, unsupervised clustering was applied using Seurat FindNeighbours and FindClusters which first connected cells together according to the “shared nearest neighbour” theory, generating a shared nearest neighbour graph and then optimised the modularity using the Louvain algorithm to identify cell clusters. The resolution parameter controlling cluster granularity was set to 0.8. Marker genes that were differentially overexpressed in each cluster compared to all other clusters were identified using Seurat FindAllMarkers with default parameters. Clusters were annotated with major lineage cell type using a combination of cluster-marker gene expression profiles and interrogating the expression of known canonical cell type marker genes.

### 2.10 NK cell cluster identification and annotation

For finer-grained analysis specifically of NK cell type, data was subset on the NK cell population identified by diminished CD3 expression along with expression of NK markers FCGR3A (CD16), GNLY, KLRF1 and NKG7, before NK-population variable features were calculated with Seurat FindVariableFeatures (n=3000 genes). This was followed by dimensionality reduction with PCA and UMAP (using PCs 1:20). A shared nearest-neighbour graph was constructed in PCA-space with Seurat FindNeighbours function and clusters are identified within this graph using Seurat FindClusters function, optimizing the modularity with the Louvain algorithm. Canonical major cell type marker gene expression profiles were assessed to remove non-NK cell clusters. Further filtering removed any remaining non-NK cells which had unique molecular identifier (UMI) counts of > 0.5 for the T cell specific CD3D gene. Final NK cell-cluster marker genes were identified with FindAllMarkers and NK differentiation marker gene expression was assessed to annotate NK cell subsets. Genes of interest panels included NK ligands, NK receptors and cytokines. Average expression of these within NK cell clusters, within the tissue types and within treatment groups were calculated using Seurat AverageExpression function. The proportions of different cell types were compared across the samples analysed, using the Wilcoxon rank sum test.

### 2.11 Module scoring

Per cell module scores were calculated for selected gene modules of interest including Cytotoxicity (GZMA, GZMB, GZMH, GZMM, GNLY, PRF1, CTSW), Exhaustion (CD39, PDCD1, TIM-3, CTLA-4), Activating Receptors (CD226, B3GAT1, KLRK1, NCR3, NCR2, NCR1, FCGR3A, KLRC2, KLRF1, CD244, CRTAM, CD27, ITGB2, CD69) and Inhibitory Receptors (TNFRSF18, KLRC1, KLRD1, KLRB1, TNFSF10, LAIR1, FAS, TIGIT, PDCD1). Scores were calculated using Seurat AddModuleScore, which calculates the average expression of the module above randomly selected background features.

### 2.12 Statistics

Statistical analysis was performed using GraphPad Prism (RRID: SCR_002798), version 9.2.0. The Mann-Whitney U and Kruskal Wallis tests were used to analyse continuous and unpaired variables, while the Chi-squared test examined categorical variables. Analysis of Variance (ANOVA) was used to compare the means between the different patient groups. Survival rates (progression free survival and overall survival) were estimated using the Kaplan Meier method, with the Mantel-cox test used to compare survival distributions. Statistical significance was regarded with p values of less than 0.05.

## 3 Results

### 3.1 Increased proportions of NK cells were observed in the serum of patients with OC and display an activated phenotype

**Figure 1:**
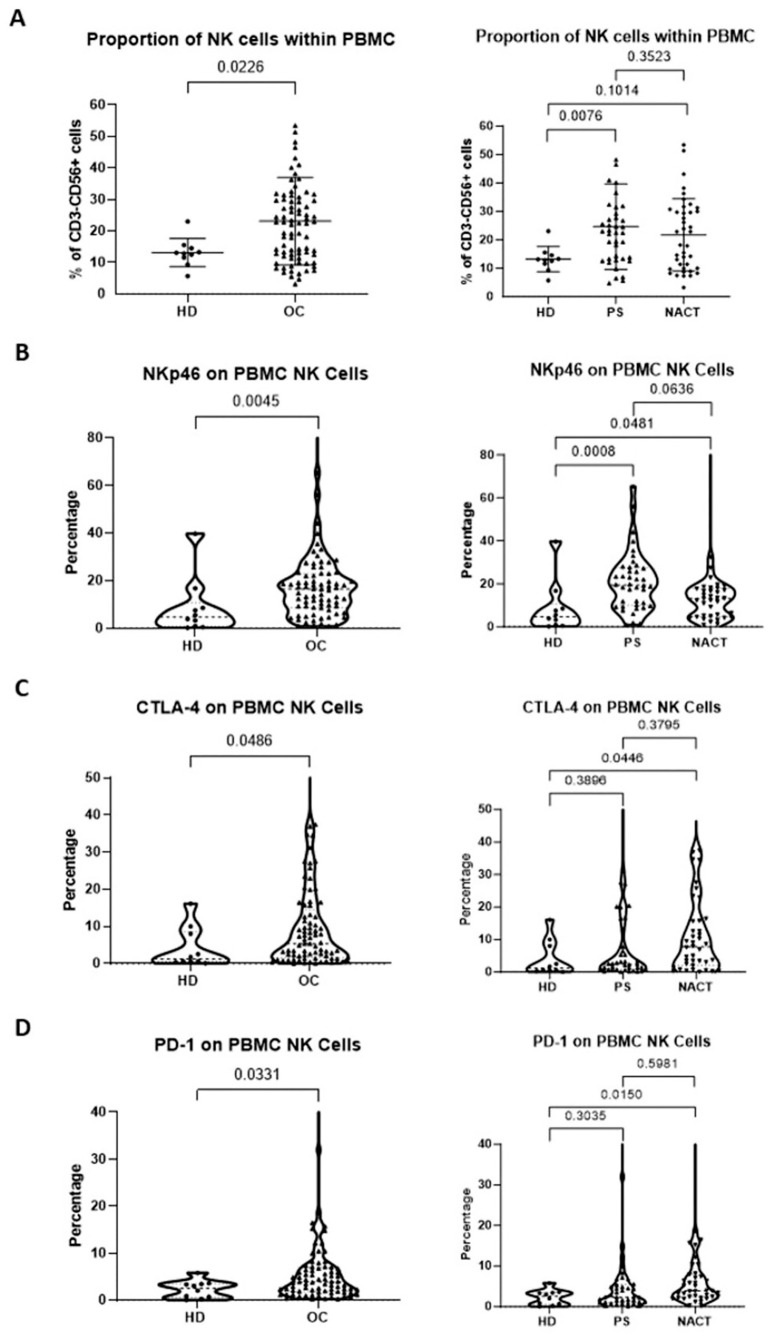
Patients with OC have an increased proportion of NK cells with activated phenotype within the peripheral blood. The proportion of peripheral blood NK cells **(A)**, percentage of NKp46 expression **(B)**, percentage of CTLA4 expression **(C)** and percentage of PD-1 expression **(D)** were compared between patients with OC (chemotherapy-naïve and chemotherapy-exposed combined) and healthy donors (HD) with normal ovaries (Left panels) and between OC patients managed with primary surgery (PS) or treated with neoadjuvant chemotherapy (NACT) and healthy donors (HD) with normal ovaries (Right panels). Data are shown as dot plot **(A)** or violin plots **(B–D)** with each dot representing a donor. The significance was determined using Mann-Whitney testing (left panels) and Kruskal-Wallis testing.

Flow cytometry was used to determine the proportion and phenotype of NK cells from the study cohort of 80 OC patients, including those treated with primary surgery (PS) and neoadjuvant chemotherapy (NACT), and compared to age-matched healthy female donors (HD) with normal ovaries. The proportion of NK cells within the CD45+ lymphocyte population was significantly greater in the peripheral blood samples from patients with OC compared to those from healthy donors (p=0.0226; median 22.7% and 12.9%, respectively; **Figure 1A** left panel). This increase was most notable in patients at the time of diagnosis prior to surgery (p=0.0076, median value 22.7%), but appeared to be attenuated in those exposed to neoadjuvant chemotherapy (p=0.1014, median value 20.5%; **Figure 1A** right panel). There was a greater proportion of peripheral NK cells with raised CD56 expression, termed CD56^bri^ NK cells, in the OC patients compared to the healthy donors (p=0.0282; median 6.4% and 2.3%, respectively). There was a similar amount of peripheral NK cells with low CD56 expression (CD56^dim^) in the OC and healthy donor cohorts (**Supplementary Figure 2**).

The total lymphocyte count in untreated OC patients was markedly reduced by 44% compared to healthy donors (p=0.0438, median value 0.2921 x 10*9/L vs 0. 5164 x 10*9/L, respectively; **Supplementary Figure 3A**) whilst the peripheral NK cell count remained comparable (p=0.8330, median value 0.0638 x 10*9/L vs 0.0628 x 10*9/L, respectively; **Supplementary Figure 3B**). This increased peripheral percentage of NK cells detected within the blood of OC patients suggests they are selectively retained compared to other lymphoid subsets.

Review of the expression of activating receptors on peripheral NK cell subsets showed increased NKp46 in OC patients, with expression seen on 17% of cells compared to 5% of healthy donors (p=0.0045; **Figure 1B** left panel). This profile was most notable in patients at presentation (median 19.4%, p=0.0008), but was also retained following NACT (median 13.2%, p=0.0481; **Figure 1B** right panel). The expression of immune checkpoint receptors PD-1 and CTLA-4 was also raised in OC patients compared to healthy donors (median value 5.4% vs 1.2% for CTLA-4, p=0.0486; median value 4.0% vs 2.5% for PD-1, p=0.0331; **Figures 1C, D**, respectively, left panels), but only reached significance after NACT (median 7.8%, p=0.0446 for CTLA-4; median 3.9%, p=0.0150 for PD-1; **Figures 1C, D** right panels).

### 3.2 Peripheral NK cells from OC patients show markedly increased cytotoxic potential but reduced levels of cytokine production

**Figure 2:**
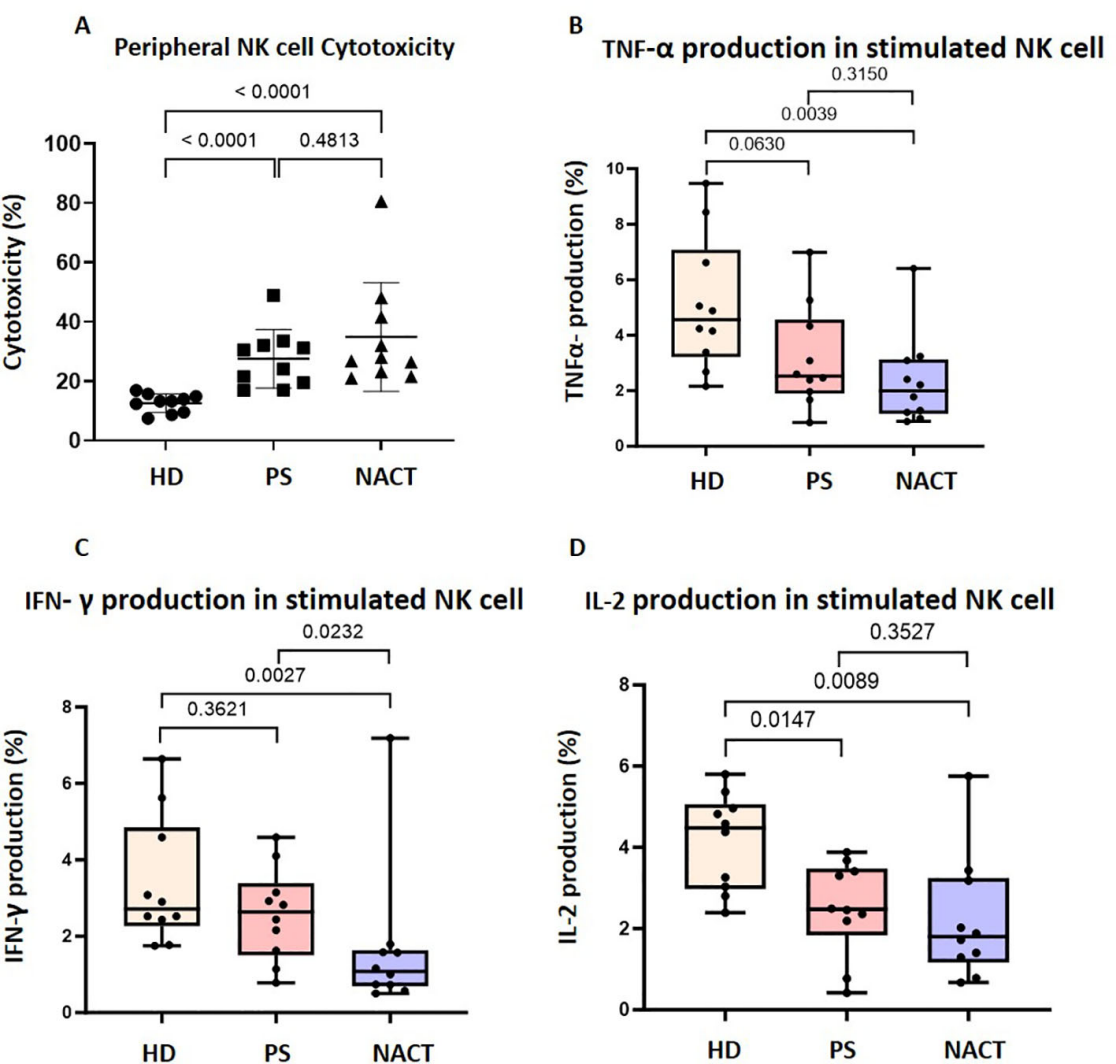
Peripheral NK cells in OC patients are cytotoxic but have impaired cytokine secretion. **(A)** The cytotoxicity of peripheral NK cells enriched from healthy donors (HD), primary surgery (PS) OC patients and neoadjuvant chemotherapy (NACT) OC patients were compared using a flow cytometry based true count assay. Data shown are as dot plots with each dot representing a donor. Whole PBMCs from OC patients and HDs were co-cultured with K562 cells and stained with **(B)** TNF- α, **(C)** IFN-γ and **(D)** IL-2 antibodies. The percentage of NK cells that produce each cytokine was compared between OC patients and HDs. Data is shown as bar charts with each dot representing a donor. The significance was determined using Kruskal-Wallis testing.

NK cell function was explored by evaluation of cytotoxicity and cytokine production in response to incubation with the K-562 target cell line. NK cells from OC patients showed a two-fold increase in cytotoxicity compared to healthy donors, with a median response of 27.3% in both PS (n=10) and NACT n=10) cohorts compared to 13.3% in healthy donors (n=10) (p< 0.0001; **Figure 2A**).

In contrast, NK cell-derived cytokine production was substantially reduced in OC patients (**Figures 2B–D**). There was a 45% decrease in IL-2 production in the PS cohort compared to the healthy donor group (p=0.0147; **Figure 2D**), while broader reductions occurred in the NACT cohort (TNF-α production decreased by 57% (**Figure 2B**), IFN-γ by 61% (**Figure 2C**) and IL-2 by 60% (**Figure 2D**)) compared to the healthy donor group (p=0.0039, p=0.0027 and p=0.0089, respectively).

### 3.3 NK cells are reduced in primary and metastatic OC tissue and exhibit downregulation of activatory receptors

**Figure 3:**
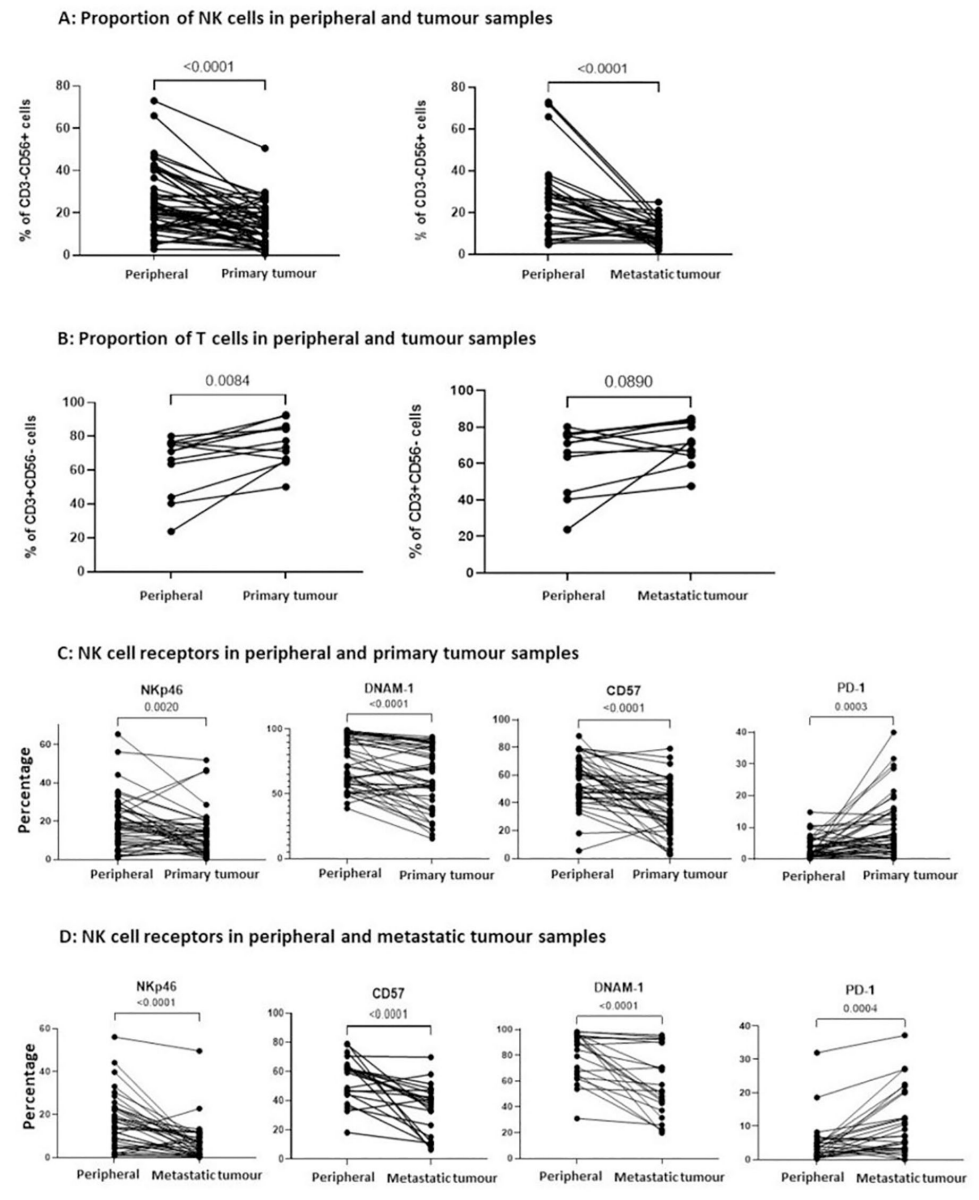
The proportion of infiltrating NK cells within primary and metastatic tumour tissue was reduced and with a less activated phenotype. **(A)** The percentage of CD3-CD56+ NK cells and **(B)** the percentage of CD3+CD56- T cells were compared between PBMCs and matched TILs from the same OC patient, displaying comparisons to primary tumour tissue (left panel) and metastatic tumour tissue (right panel). The percentage of NKp46, CD57, DNAM-1 and PD-1 expression on NK cells was compared between PBMCs and TILs from either primary tumour tissue **(C)** or metastatic tumour tissue **(D)** from OC patients. Data are shown as dot plots with each dot representing a matched donor. The significance was determined using Wilcoxon test.

Comparison of primary ovarian tumour and metastatic omental tissue within the same patient revealed similar proportions of NK cells at the tissue sites, which were both lower than the proportion within the peripheral blood: 17% and 12% in primary tumour and metastatic tissue, respectively, compared to 23% in blood (**Figure 3A**). In contrast, the proportion of T cells within the primary tumour was greater compared to blood (75.8% and 63.7%, respectively), whilst values in metastatic tumour and blood were comparable (**Figure 3B**).

The expression of activatory receptors NKp46, DNAM-1 and CD57 was decreased on NK cells in primary tumour compared to blood (NKp46: 9.8% on primary tumour NK cells vs 18.2% on peripheral NK cells, p=0.0020; DNAM-1: 59.7% vs 71.7%, P<0.0001; CD57: 38.9% vs 59.6%, p<0.0001). In contrast, the inhibitory checkpoint PD-1 was expressed on 7.4% of tumour NK cells, a two-fold increase compared to the NK cells within blood (p=0.0003; **Figure 3C**). Similar expression correlations were evident between the NK cells within metastasis and blood (NKp46: 5.3% on metastatic NK cells vs 14.8% on peripheral NK cells, p<0.0001; DNAM-1: 51.9% vs 89.3%, p<0.0001; CD57: 37.6% vs 61.1%, p<0.0001), whilst elevated PD-1 expression on 6.9% of cells was observed (p=0.0004; **Figure 3D**). This profile of reduced expression of activation receptors together with increased levels of immune checkpoints indicate that the cytotoxic potential of NK cells within OC tumour tissue is likely to be impaired.

Upon comparison of the NK cells in the patients without ovarian tumours, there were similar proportions of NK cells in the peripheral blood and both matched normal ovarian tissue (p=0.2188) and matched normal omental tissue (p=0.4385). There were also no differences in the expression of NK cell receptors between the blood and tissue in the non-malignant cases.

### 3.4 NK cells in metastatic tumour show features of reduced cytotoxicity and increased exhaustion which associate with disease recurrence

**Figure 4:**
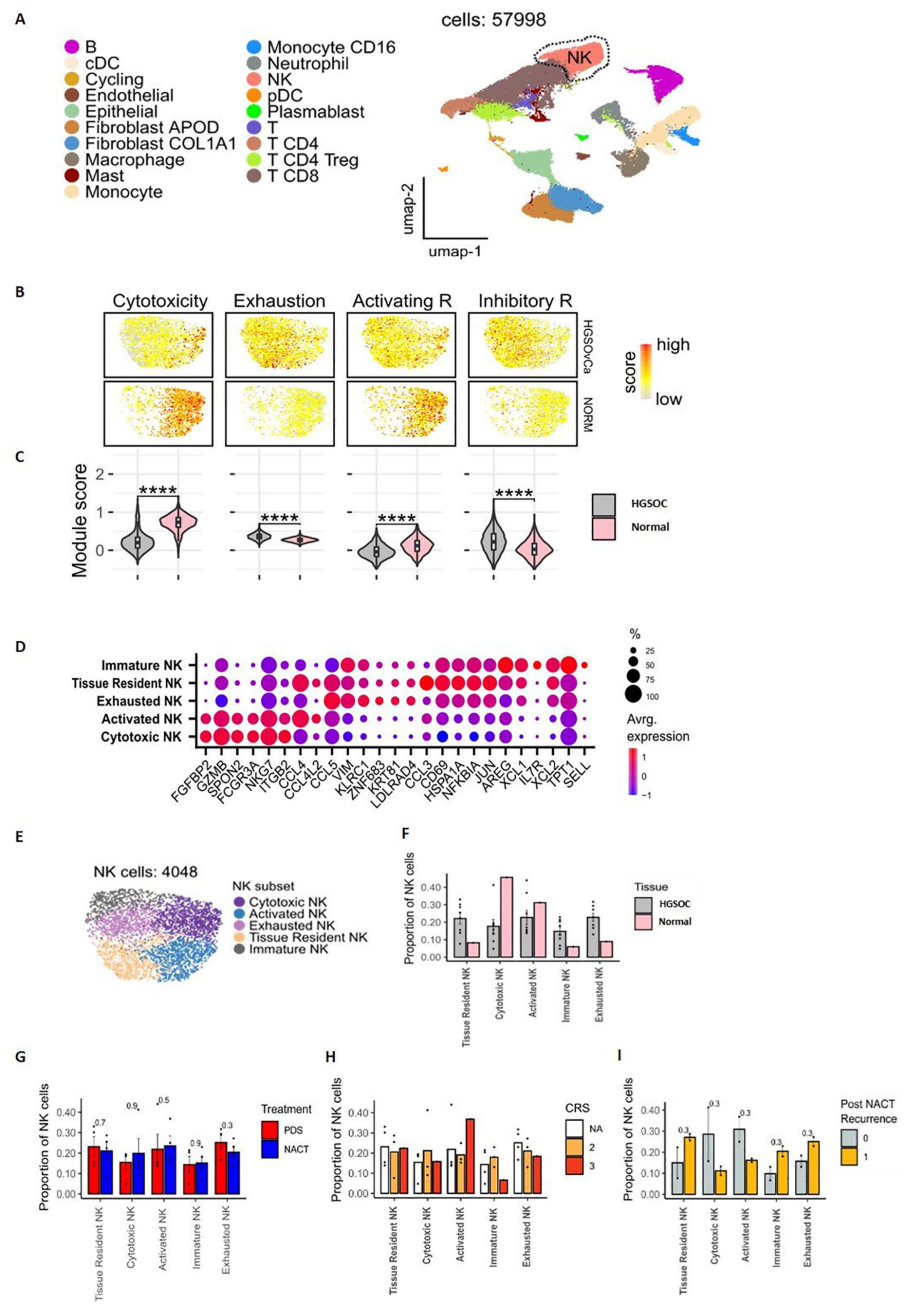
Transcriptional characterisations of NK cells within omental tissue. UMAP embedding of integrated scRNAseq data overlaid with major lineage cell type clusters **(A)**. UMAP embeddings of NK cells overlaid with signature module scores for NK cytotoxicity, exhaustion, activating receptors and inhibitory receptors to identify the function of NK cells **(B)**. The distributions of module scores stratified by tissue type, comparing omental tumour (n=8) and normal omentum (n=1) in patients with HGSOC **(C)**. Dot plot to show the average expression of the top marker genes identified as being overexpressed in different NK subpopulations. The size of the dots indicates the proportion of cells within the cluster that are expressing the given gene **(D)**. UMAP embedding of NK cell populations overlaid with the NK cell subpopulations identified by unsupervised clustering **(E)**. The proportion of NK cell subtypes in omental tumour (n=8) and normal omental tissue (n=1) **(F)**, prior to chemotherapy (PDS cohort) vs following neoadjuvant chemotherapy (NACT cohort) **(G)** and stratified by Chemotherapy Response Score (CRS). NA = chemotherapy-naïve, primary surgery samples **(H)**. The proportion of NK cell clusters in patients who had disease recurrence 18 months following chemotherapy treatment (1) compared to those that remained disease free (0) **(I)**.

We used scRNA-seq analysis to assess the transcriptome and heterogeneity of NK cells within metastatic omental tissue from 9 women with stage III/IV HGSOC (**Table 2**). Of these, 5 patients were chemotherapy-naive (PDS) whilst 4 had received NACT. Tumour was confirmed in 8 patients, with no evidence of metastatic disease in 1 patient. This normal omental sample was included in the analysis for comparison to the patients with omental tumours in the PDS and NACT groups. Unsupervised clustering and canonical lineage marker genes identified major lineage cell types (**Figure 4A**; **Supplementary Figure 4**). The NK cell population was identified by high expression of *PTPRC* (protein tyrosine phosphatase, receptor type C) encoding CD45, diminished CD3, and the expression of NK marker genes GNLY, KLRF1, FCGR3A (CD16) and *NKG7*, a regulator of NK cytotoxicity. Data was subset on NK cells only and selected gene module scores compared between metastasis and normal tissue (**Figures 4B, C**), showed downregulation of cytotoxicity genes within malignant disease together with increased expression of gene modules for NK exhaustion and inhibitory receptors. The expression profile of NK cell ligands was also examined (**Supplementary Figure 5**) and here it was notable that non-haemopoietic epithelial, endothelial and fibroblast cells typically demonstrated the potential for inhibitory NK cell engagement. In contrast, potential activatory NK cell engagement was seen to derive mainly through lymphoid and myeloid subsets.

**Table 2:** Characteristics of HGSOC patients utilised for scRNAseq analysis.

Variable	Primary surgery (n=4)	Neoadjuvant chemotherapy (n=4)	Normal omentum (n=1)
**Age** (years) Mean ± SD	60.0 ± 12.1	66.3 ± 8.2	72
**Ethnicity** White Asian Black	**n (%)** 3 (75.0) 0 (0.0) 1 (25.0)	**n (%)** 2 (50.0) 1 (25.0) 1 (25.0)	**n (%)** 1 (100.0) 0 (0.0) 0 (0.0)
**Disease stage** 3 4	**n (%)** 3 (75.0) 1 (25.0)	**n (%)** 1 (25.0) 3 (75.0)	**n (%)** 0 (0.0) 1 (100.0)
**PCI** Median (range)	21 (14-25)	16 (12-18)	6
**BRCA status** normal pathological variant	**n (%)** 3 (75.0) 1 (25.0; BRCA1)	**n (%)** 4 (100.0) 0 (0.0)	**n (%)** 1 (100.0) 0 (0.0)
**CA-125** (U/ml) Mean ± SD	898 ± 1398	2010 ± 1968	2013
**Cytoreduction** R0 R1 R2	**n (%)** 1 (25.0) 1 (25.0) 2 (50.0)	**n (%)** 3 (75.0) 0 (0.0) 1 (25.5)	**n (%)** 1 (100.0) 0 (0.0) 0 (0.0)
**Chemotherapy cycles** 3 4 6	n/a	**n (%)** 1 (25.0) 2 (50.0) 1 (25.0)	n/a
**Chemotherapy Response score** 2 3	n/a	**n (%)** 3 (75.0) 1 (25.0)	n/a
**Disease recurrence** Recurred Disease-free	**n (%)** 1 (25.0) 3 (75.0)	**n (%)** 2 (50.0) 2 (50.0)	**n (%)** 0 (0.0) 1 (100.0)
**Survival** Died Alive	**n (%)** 1 (25.0) 3 (75.0)	**n (%)** 2 (50.0) 2 (50.0)	**n (%)** 0 (0.0) 1 (100.0)

Unsupervised clustering identified 5 NK subpopulations with functional subtypes allocated based on differential expression of marker genes (**Figures 4D, E**). A cytotoxic NK subset had high expression of cytotoxicity genes *NKG7, FGFBP2, GZMB* and *FCGR3A*, while an activated NK subset had raised activation markers *CCL4* and *CCL4L2*. An exhausted NK subset had low levels of *GZMB* and *CD16* together with raised expression of inhibitory markers *KLRC1* and *GZMK*. Tissue resident (increased *CCL4, CCL3, JUN, HSPA1A* and *CD69* genes) and immature (increased *IL7R, TPT1, AREG* and *XCL1/2* genes) NK clusters were also seen. Notably, the cytokine expression profile across NK subsets was also highly distinct (**Supplementary Figure 6**).

Expression profile of activating and inhibitory NK receptors showed DNAM-1 to be more dominant in cytotoxic and activated subsets (**Supplementary Figure 7A**) whilst inhibitory receptors KLRC1 and TIGIT were expressed in exhausted and tissue resident NKs. The subsets with elevated DNAM-1 had a differing gene expression profile to the subsets with low DNAM-1, specifically with upregulation of FGFBP2, KLRF1 and GZMB/H with increasing DNAM-1 (**Supplementary Figure 7B**). A pseudotime analysis (**Supplementary Figure 7C**), infers the likely lineage trajectory of the NK cells, with the immature NK subset as the source population, which gives rise to the DNAM-1 expressing cytotoxic and activated clusters.

Comparison between normal and metastatic tissue showed greater proportions of cytotoxic and activated NK subpopulations in normal tissue whilst exhausted, immature and tissue resident NK cell subpopulations were increased within tumour (**Figure 4F**). An in-depth cellular interaction analysis explored the cell signalling received and sent by the NK cell subpopulations and the other cell populations (**Supplementary Figure 8**). The intra-tumoural NK cells predominantly interacted with the Fibroblast cell populations.

NK cell clusters were then evaluated according to chemotherapy exposure, and the degree of pathological and clinical response to chemotherapy. Chemotherapy-naïve patients had an increased proportion of exhausted NK cells compared to the those who had received chemotherapy (**Figure 4G**). A trend was observed towards a greater proportion of activated NK cells and less immature and exhausted NK cells with complete response to chemotherapy (CRS3) compared to patients with moderate responses (CRS 2) (**Figure 4H**). The NK profile was next assessed in relation to disease recurrence (n=2) or disease-free status (n=2) following chemotherapy. Higher proportions of exhausted, immature and tissue resident NK cells were seen in patients with disease recurrence whilst higher levels of activated and cytotoxic NK cells associated with disease free survival (**Figure 4I**).

### 3.5 Increased DNAM-1 expression is seen on NK cells in late-stage disease and correlates with poor outcome, although DNAM-1 mediated cytotoxicity of ovarian cells is observed *in vitro*

**Figure 5:**
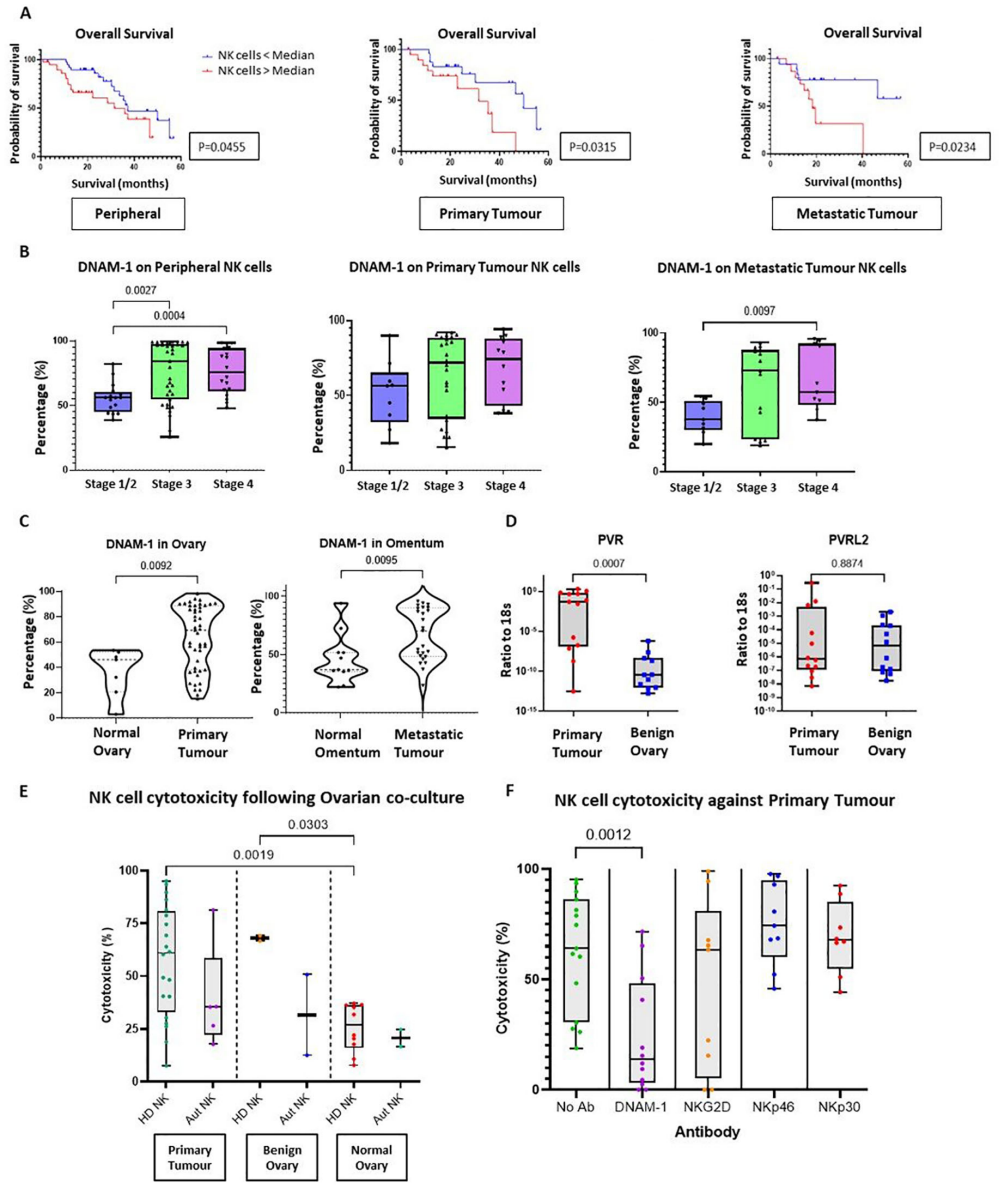
The importance of activating receptor DNAM-1 in Ovarian Cancer. **(A)** Kaplan-Meier curve (log-rank test) to compare the overall survival of HGSOC patients between two groups according to the percentage of DNAM-1 expression on NK cells (cut-off point as median DNAM-1 expression) from whole PBMCs (left panel), TILs from either primary tumour tissue (middle panel) or metastatic tumour tissue (right panel). **(B)** Bar charts to compare the percentage of DNAM-1 expression on NK cells from whole PBMCs (left panel), TILs from either primary tumour tissue (middle panel) or metastatic tumour tissue (right panel) from OC patients according to disease stage. Data are shown as bar charts with each dot representing a donor. Significance was determined using Kruskal-Wallis testing. **(C)** Violin plots to compare the percentage of DNAM-1 expression on NK cells from tissue between normal ovary versus primary ovary tissue (left panel) and normal omental tissue versus metastatic omental tumour tissue (right panel). Data are shown as violin plots with each dot representing a donor. Significance was determined using Mann Whitney testing. **(D)** Total RNA extracted from OC tissue and benign ovary for Q-PCR to detect the ratio of copy numbers of PVR and PVRL2 to 18s. Data shown as bar charts with each dot representing a donor. Significance determined using Mann Whitney testing. **(E)** The cytotoxicity against primary OC tissue, benign ovarian tissue or normal ovarian tissue using peripheral NK cells enriched from either HD or autologous PBMCs. Data shown as bar charts with each dot representing a donor. The significance was determined using Mann Whitney testing. **(F)** Cytotoxicity assay with primary OC tissue and enriched allogenic PBMCs NK cells in the presence of no antibodies, anti-DNAM-1, anti-NKG2D, anti-NKp46 or anti-NKp30 antibodies. Data are shown as bar charts with each dot representing a donor. Significance was determined using Mann Whitney testing.

The pattern and clinical correlates of NK cell activatory or inhibitory receptor expression within tumour was next examined. Having identified reduced NK activatory receptors NKp46, DNAM-1 and CD57 in tumour tissue compared to blood (**Figures 3C, D**), the expression patterns were correlated to clinical outcomes. The mean follow-up duration of the 80 OC patients within the cohort was 30 months (range 6-60 months) during which time 36 patients (45%) had died from OC. Of the 80 OC patients, no association was observed between NK receptor expression and disease recurrence or mortality apart from decreased overall survival (OS) in patients with expression of DNAM-1 above the median value (**Table 3**; **Figure 5A**). This was seen consistently in relation to expression on NK cells in blood, primary tumour and metastatic samples (p=0.0455, p=0.0315 and p=0.0234, respectively). The determinants and correlates of the DNAM-1 receptor were therefore explored in more detail.

**Table 3:** Overall survival of HGSOC patients based on the expression of NK cell receptors.

	Overall survival	
NK cell receptor	Receptor expression above median	P Value
PBMC NK (n=80):		
*DNAM-1*	**Decreased OS**	**0.0455**
*NKp46*	No difference	0.9223
*CD57*	No difference	0.0825
*PD-1*	No difference	0.6001
Ovarian NK (n=49):		
DNAM-1	**Decreased OS**	**0.0315**
NKp46	No difference	0.0972
CD57	No difference	0.4267
PD-1	No difference	0.641
Omental NK (n=39):		
DNAM-1	**Decreased OS**	**0.0234**
NKp46	No difference	0.5627
CD57	No difference	0.0612
PD-1	No difference	0.66

OS, overall survival.

DNAM-1 expression was next interrogated in relation to disease stage (**Figure 5B**) and increased expression was seen on blood NK cells in both stage III and IV disease (p=0.0027 and p=0.0004, respectively), together with increased expression within tumour in stage IV metastasis (p=0.0097). DNAM-1 was expressed highly on infiltrating NK cells in both primary and metastatic tumour compared to normal ovarian and omental tissues (p=0.0092 and p=0.0095, respectively) (**Figure 5C**). As such, transcriptional expression of DNAM-1 ligands was determined using qRT-PCR (**Figure 5D**) and revealed PVR (CD155) to be markedly increased in HGSOC tissue compared to benign ovarian biopsies.

Finally, we investigated the importance of the DNAM-1 signalling pathway in NK cell-mediated cytotoxicity towards HGSOC. This was assessed using co-cultures of digested single cells from ovarian tissue (including primary HGSOC, benign =and normal ovary cultures) together with either autologous or allogenic NK cells. The average cytotoxicity of allogeneic donor NK cells towards advanced OC tumour cells was 57.1% (range of 18.8% to 95.1%) while autologous NK cells displayed comparable cytotoxicity of 39.3% (range of 17.7% to 81.3%) (**Figure 5E**). Notably, NK cytotoxicity towards HGSOC cells was similar to that seen in benign ovarian co-cultures (average cytotoxicity of 68.0% and 31.6% for allogeneic and autologous NK cells, respectively), showing that benign ovarian cells are also sensitive to NK cell cytotoxicity Interestingly, NK cytotoxicity was reduced when co-cultured with normal ovarian tissue (average of 25.6% and 20.7% for allogeneic and autologous NK cells, respectively). Compared with NK cytotoxicity towards HGSOC, the killing by healthy donor allogeneic NK cells was significantly lower towards the normal ovarian cells (p=0.0019).

The co-cultures were repeated in the presence of blocking antibodies against the NK receptors DNAM-1, NKp30, NKp46 and NKG2D (**Figure 5F**), using allogenic NK cells due the limited availability of autologous cells. Mean cytotoxicity was reduced from 62% to 11% with DNAM-1 blockade (p=0.0012) whilst no consistent changes were observed for blockade of NKG2D, NKp46 or NKp30.

## 4 Discussion

The phenotype and clinical importance of NK cells in human tumours remains uncertain, but of substantial interest. Here we have interrogated a large cohort of 80 patients with ovarian cancer in the most comprehensive study of NK cells to date. The findings reveal a range of novel features that support the critical role of NK function in clinical outcome.

Our work initially studied NK cells within blood where typically NK cells account for 10-15% of circulating lymphoid cells (18). Strikingly, this proportion was increased nearly two-fold in patients to reach 23%, a feature that has been seen in some other tumour subtypes (32, 33). However, the absolute NK cell count was comparable to healthy donors, revealing that the increased circulating proportion reflects a differential reduction in other lymphoid cells, most likely representing migration of T cells into tumour tissue. T cells are important mediators of tumour-specific immunity and these features suggest that suppression of NK cell migration into the tumour microenvironment may contribute to tumour evasion from NK cell recognition (12, 32–35).

Despite the relative maintenance of NK cell numbers within peripheral blood in tumour patients it was also clear that these cells displayed a range of phenotypic differences from control subjects. In particular, high levels of cytotoxicity and elevated levels of NKp46 were seen in combination with reduced cytokine production, indicating a highly activated NK phenotype with functional features suggestive of late differentiation (21, 22). High expression of the checkpoint receptors PD-1 and CTLA4 was also apparent, most notably after chemotherapy. The underlying basis for this maturation of peripheral phenotype is unclear but could include a response to systemic mediators released by tumour or immune tissue. A differential production of NK cells from the bone marrow might also contribute to this effect and there was a notable increased proportion of CD56^bri^ NK cells, typically reflecting an immature phenotype.

A primary focus was on the profile of NK cells within tumour and here we observed a range of features indicating functional suppression and exhaustion, a profile seen previously in many tumours (17, 19, 36–40). ScRNA seq analyses have been applied to analyse NK cells in the Pan-cancer manner, and great heterogeneity of NK cell composition in different cancers has been observed (41). Our scRNA-seq transcriptional analysis from metastatic tissue allowed delineation of distinct NK cell subpopulations and extended prior findings with the identification of 5 distinct clusters (42–44). Increased proportions of exhausted, immature and inhibited cells were seen in tumour tissue and likely underpin previous observations of NK dysfunction (45–47).

Despite chemotherapy not altering the NK subpopulations, a complete treatment response (CRS 3) was associated with a greater proportion of activated NK cells and reduced immature NK cells. In addition, the NK cells in those who remained disease free after chemotherapy demonstrated greater cytotoxicity and activation. Chemotherapy therefore appears to reverse the degree of tumour-mediated NK inhibition and to promote NK activation, a profile that has also been seen in other tumours (48). Transcriptional analysis of paired pre and post-chemotherapy OC samples has also shown NK cell expansion, cytotoxic NK cell infiltration into tumour and increased cytotoxic gene expression following neoadjuvant chemotherapy (49).

Our study identified NK cell expression of DNAM-1 as an important determinant of clinical progression and the importance of this ligand in OC has been previously observed (50–52). Expression was increased on tumour-infiltrating NK cells whilst the PVR ligand was also significantly upregulated in tumour tissue. Carleston et al. demonstrated that the interaction between DNAM-1 and PVR is critical for NK activation and tumour killing using peritoneal effusion samples in OC (50). In addition, NK cell recognition of OC cells is increased following blockade of TIGIT which is an inhibitory receptor that competes with DNAM-1 for binding to target ligands (51).

A striking finding was a strong association between DNAM-1 expression and clinical stage, with increased levels of DNAM-1 on NK cells in patients with advanced OC or high tumour burden. DNAM-1-specific tumour cytotoxicity was observed *in vitro* and it is likely that NK cell function is suppressed within the immunosuppressive tumour microenvironment. Inflammation can also act to drive tumour progression but at this stage there is no evidence that DNAM-1-mediated NK cell engagement acts to support tumour growth.

Although our findings are likely relevant to HGSOC (given just 7 samples were of alternative histology), our results add to the understanding of how local and systemic NK cell suppression may contribute to the development and metastasis of OC. A range of potential tumour escape mechanisms were revealed, including NK cell exclusion together with an imbalance in the relative expression of activatory and inhibitory receptors. The increased activation status of NK within blood may reflect recent egress from tumour or the influence of systemic tumour-derived mediators. The potential ability of the tumour to modulate bone marrow production and maturation of NK cells could also be important. Nevertheless, patients demonstrated a high rate of disease metastasis and as such this NK cell phenotype, including increased expression of inhibitory receptors PD-1 and CTLA-4, is clearly suboptimal in the control of disease progression (12, 53). An apparent paradox was the observation that DNAM-1 expression on NK cells correlated with poor clinical outcome whilst DNAM-specific lysis of tumour tissue was apparent *in vitro*. We propose that high levels of PVR expression with advancing disease act to select for DNAM-1 upregulation but that cellular cytotoxicity is suppressed in the tumour microenvironment. This offers the potential for new approaches for OC immunotherapy, possibly acting through regulation of other members within the TIGIT protein family or DNAM-1 CAR-NK therapy (54).

## Data Availability

Single cell RNA sequencing data presented in the study are deposited in the Gene Expression Omnibus, GEO accession number GSE281120.
