# Supplementary material for: The emergence of DNAM-1 as the facilitator of NK cell-mediated killing in ovarian cancer
Source: Front Immunol. 2025 Jan 6;15:1477781. doi: 10.3389/fimmu.2024.1477781 (PMC11743932; doi:10.3389/fimmu.2024.1477781)
Supplement: Supplementary file 1 [file Presentation1.pptx]

## Slide 1
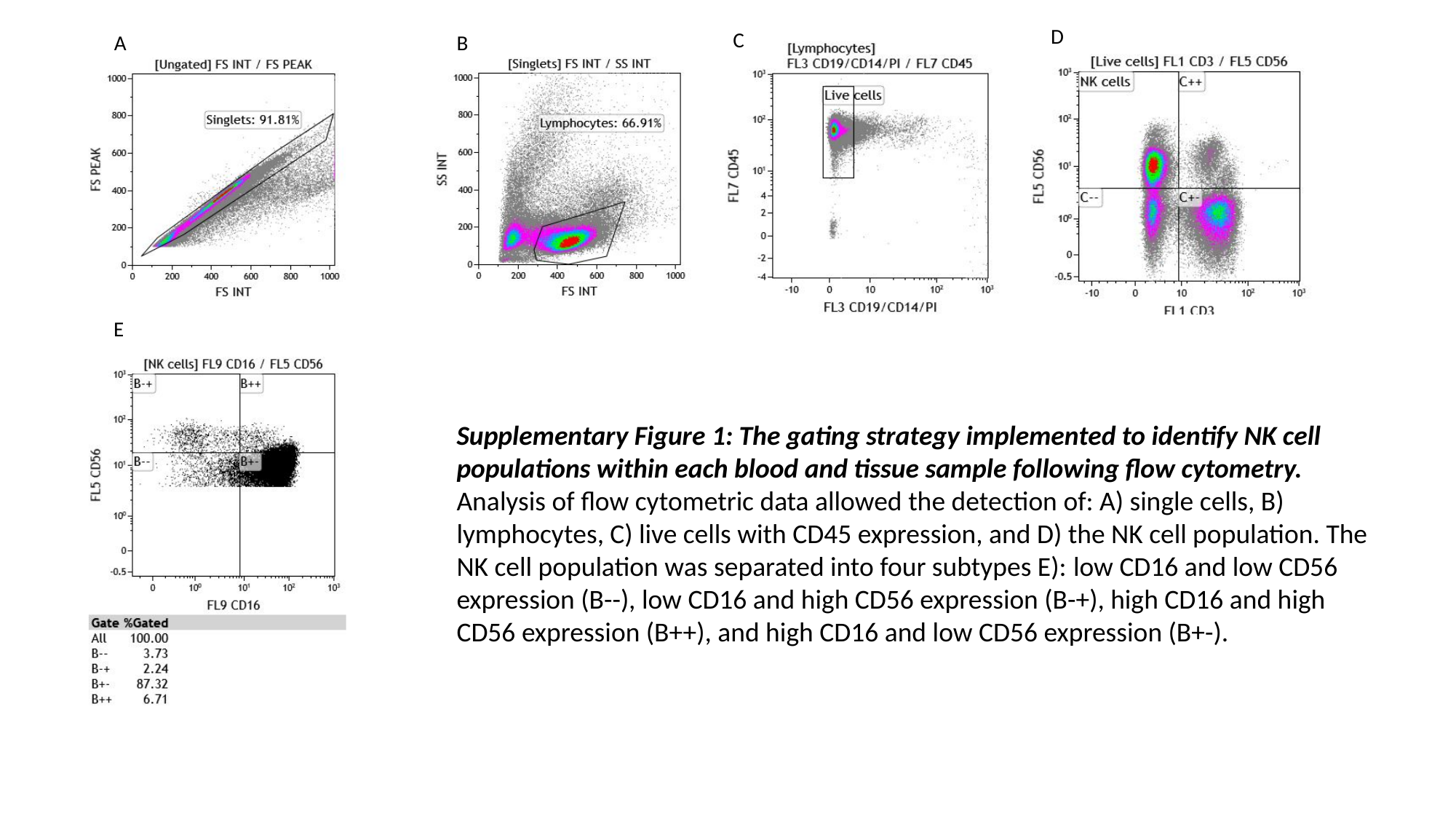

D
C
A
B
E
Supplementary Figure 1: The gating strategy implemented to identify NK cell populations within each blood and tissue sample following flow cytometry.
Analysis of flow cytometric data allowed the detection of: A) single cells, B) lymphocytes, C) live cells with CD45 expression, and D) the NK cell population. The NK cell population was separated into four subtypes E): low CD16 and low CD56 expression (B--), low CD16 and high CD56 expression (B-+), high CD16 and high CD56 expression (B++), and high CD16 and low CD56 expression (B+-).

## Slide 2
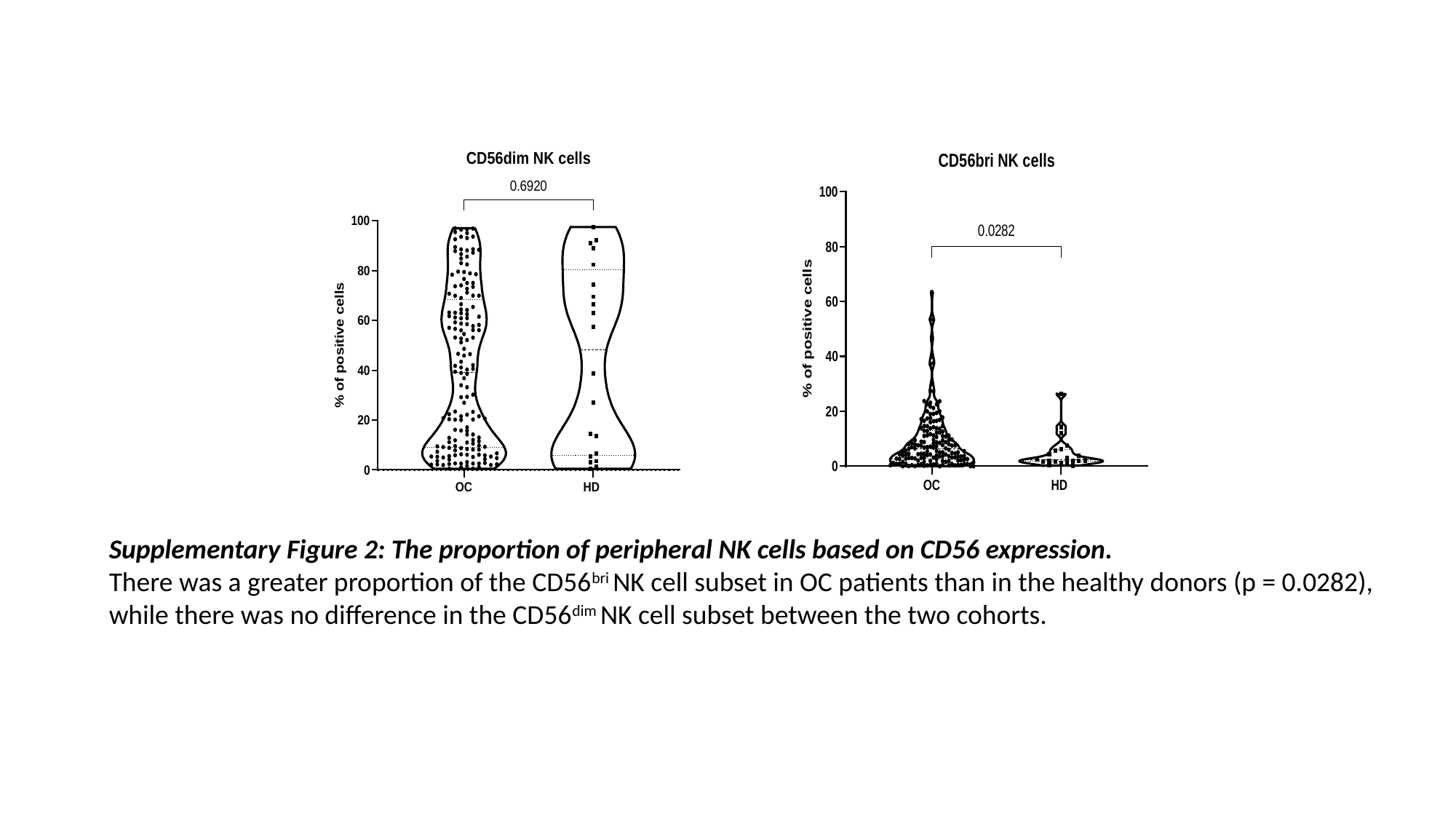

Supplementary Figure 2: The proportion of peripheral NK cells based on CD56 expression.
There was a greater proportion of the CD56bri NK cell subset in OC patients than in the healthy donors (p = 0.0282), while there was no difference in the CD56dim NK cell subset between the two cohorts.

## Slide 3
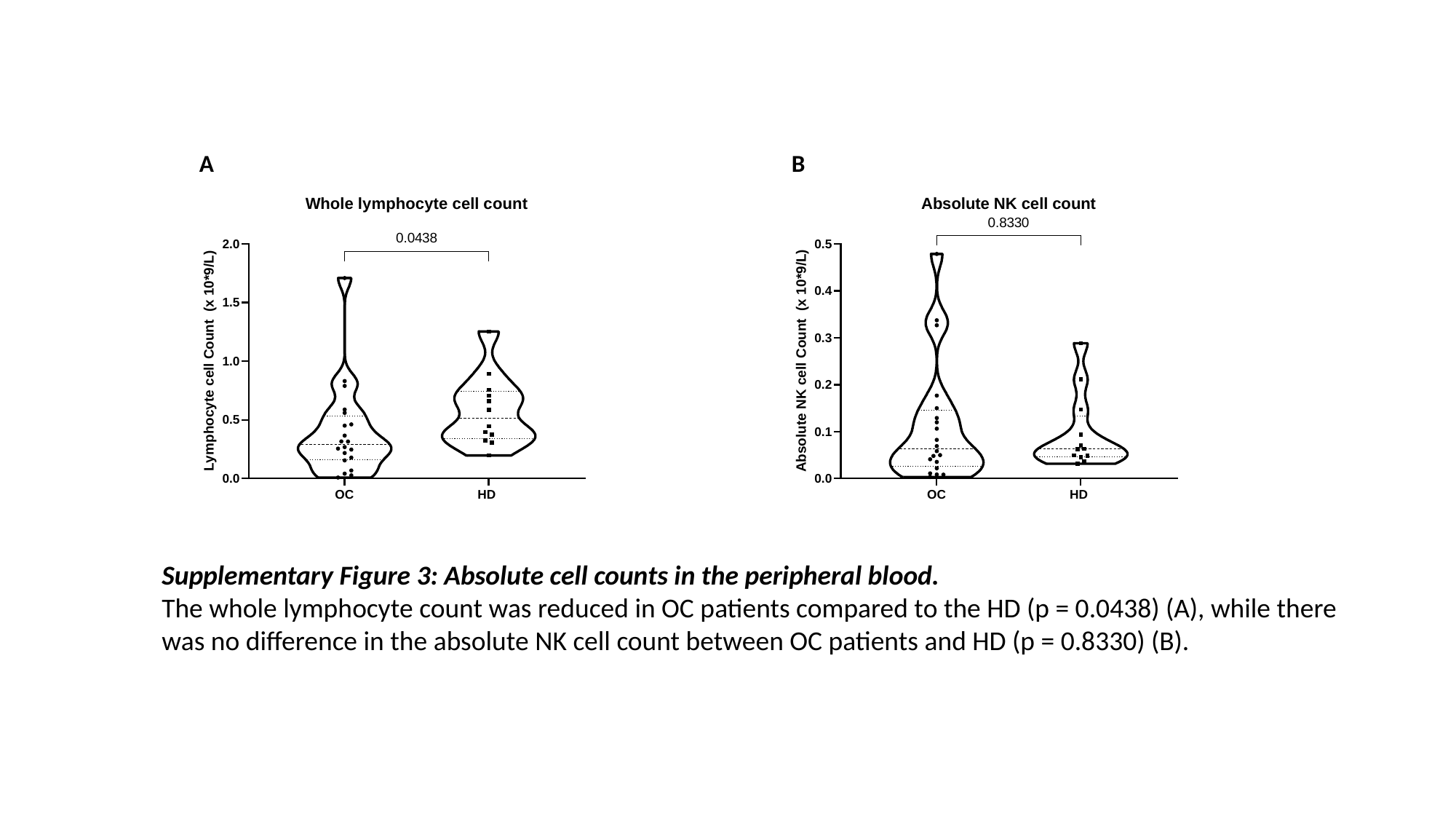

A
B
Supplementary Figure 3: Absolute cell counts in the peripheral blood.
The whole lymphocyte count was reduced in OC patients compared to the HD (p = 0.0438) (A), while there was no difference in the absolute NK cell count between OC patients and HD (p = 0.8330) (B).

## Slide 4
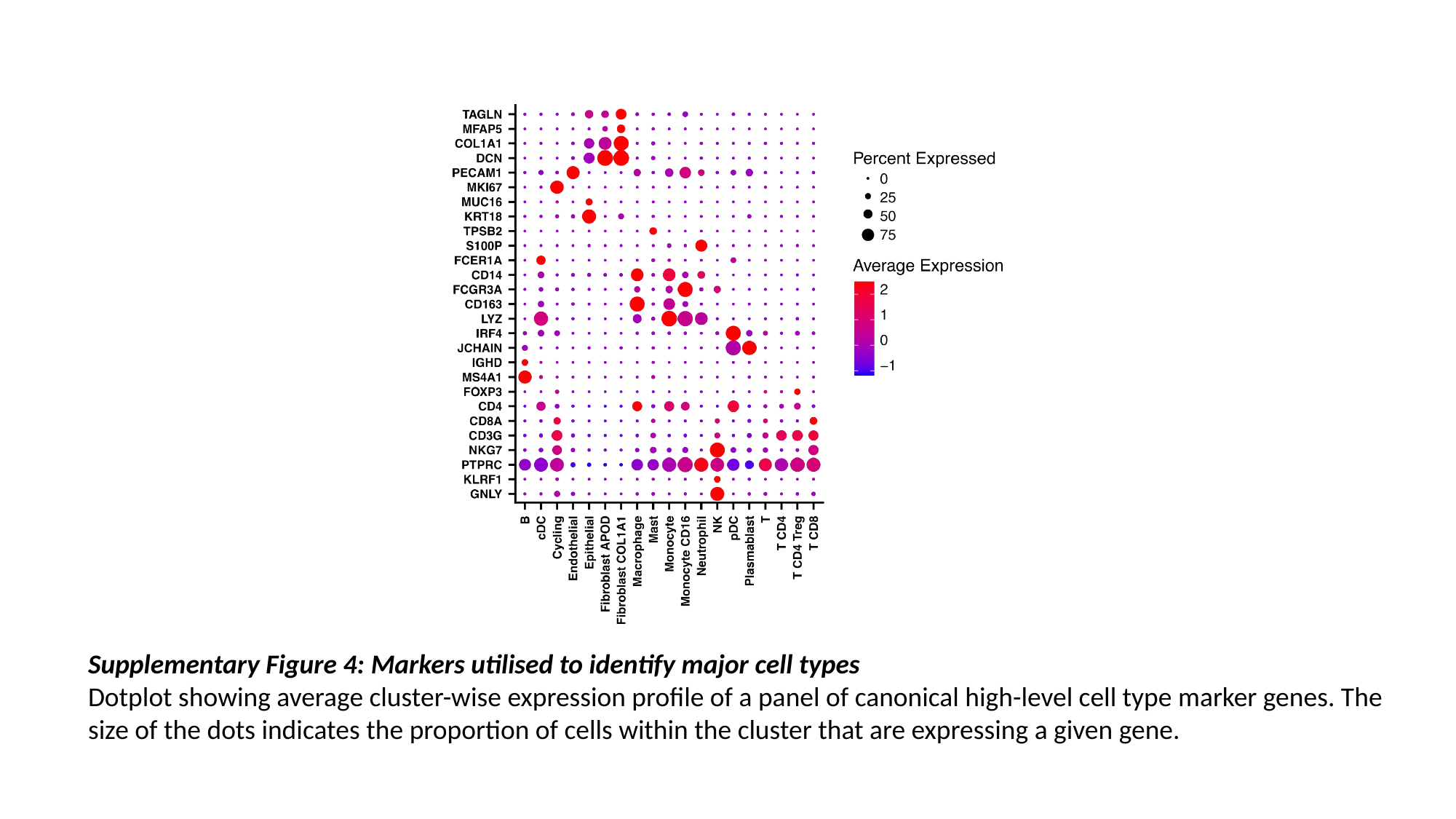

Supplementary Figure 4: Markers utilised to identify major cell types
Dotplot showing average cluster-wise expression profile of a panel of canonical high-level cell type marker genes. The size of the dots indicates the proportion of cells within the cluster that are expressing a given gene.

## Slide 5
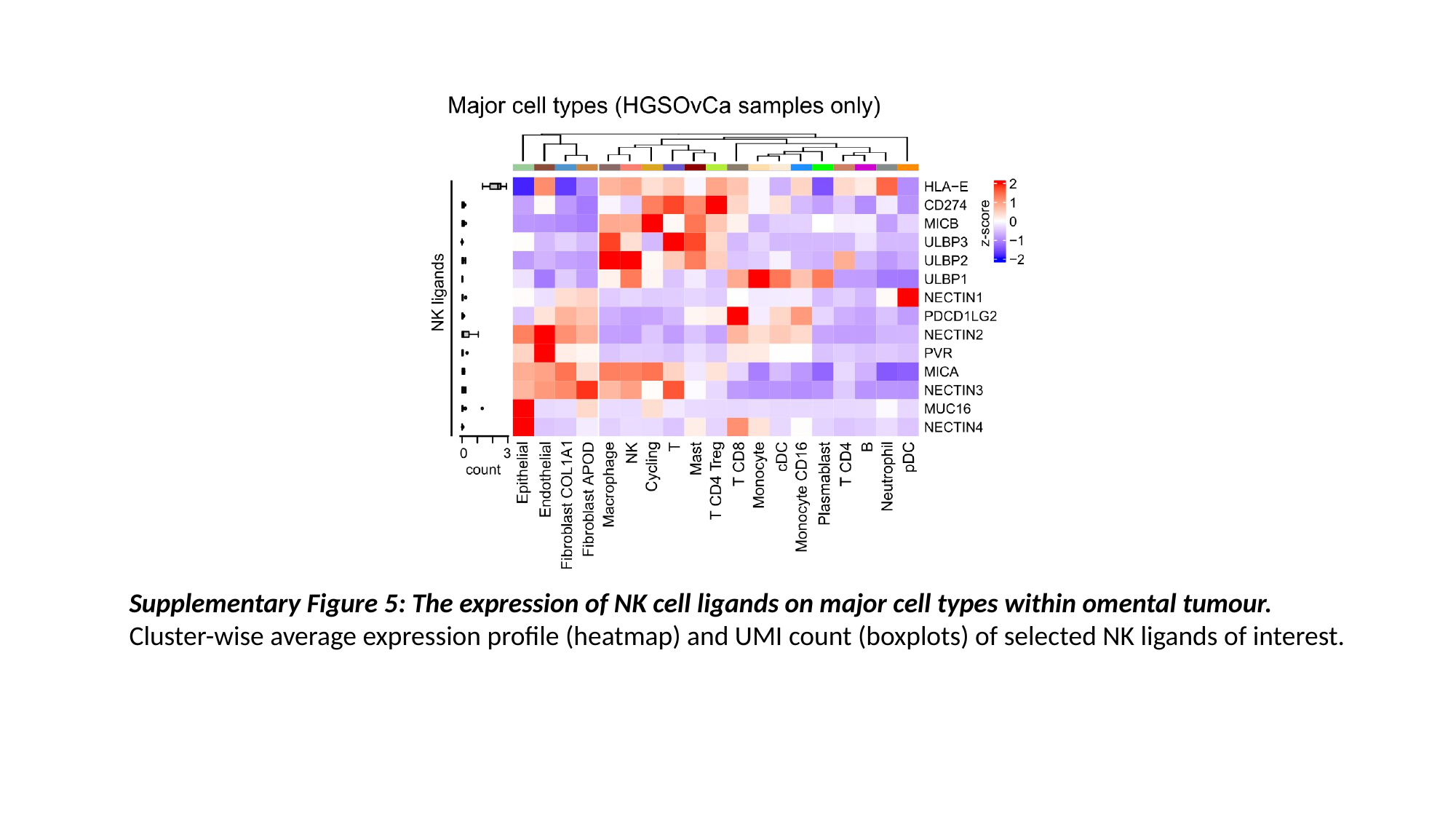

Supplementary Figure 5: The expression of NK cell ligands on major cell types within omental tumour.
Cluster-wise average expression profile (heatmap) and UMI count (boxplots) of selected NK ligands of interest.

## Slide 6
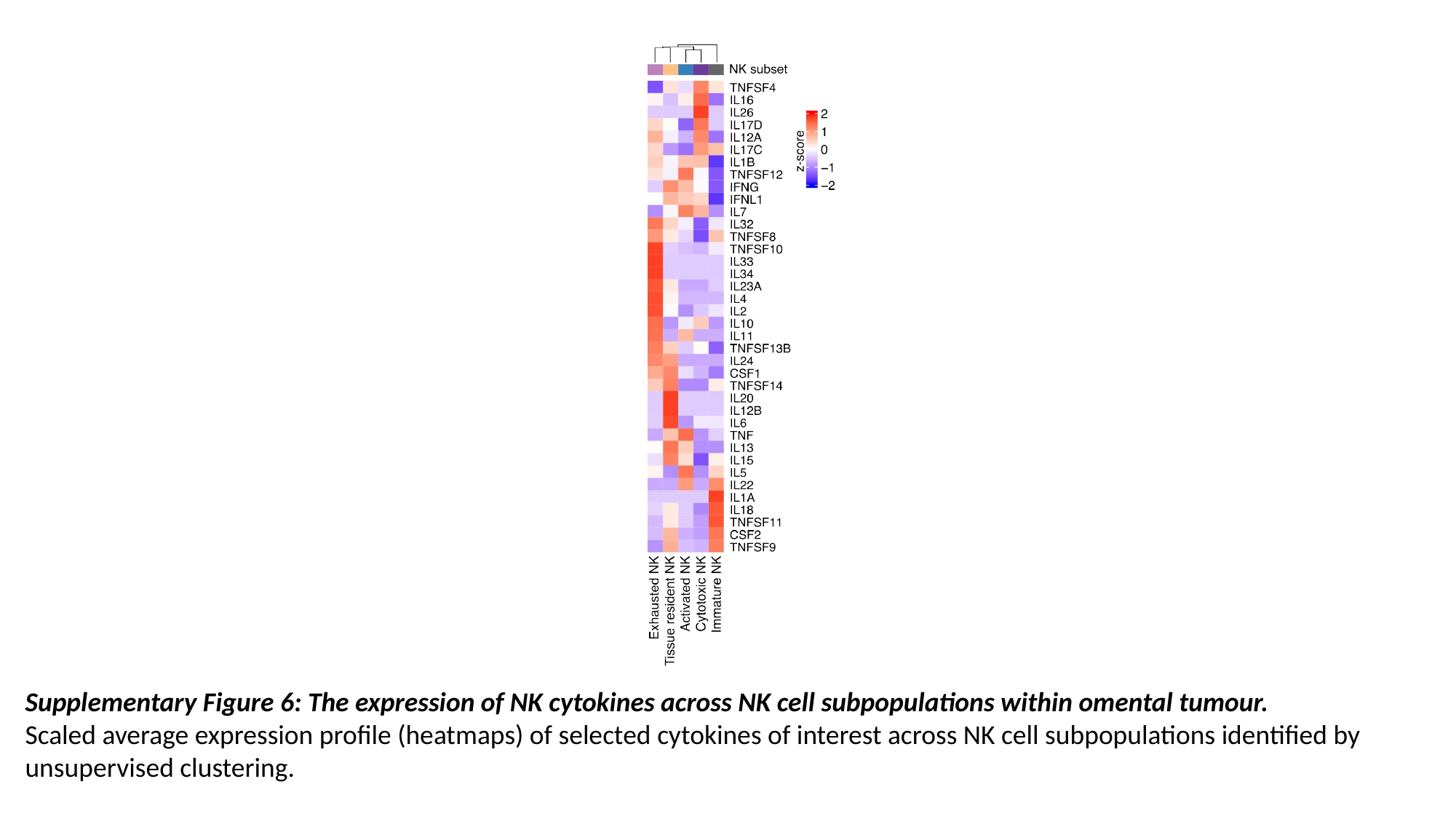

Supplementary Figure 6: The expression of NK cytokines across NK cell subpopulations within omental tumour.
Scaled average expression profile (heatmaps) of selected cytokines of interest across NK cell subpopulations identified by unsupervised clustering.

## Slide 7
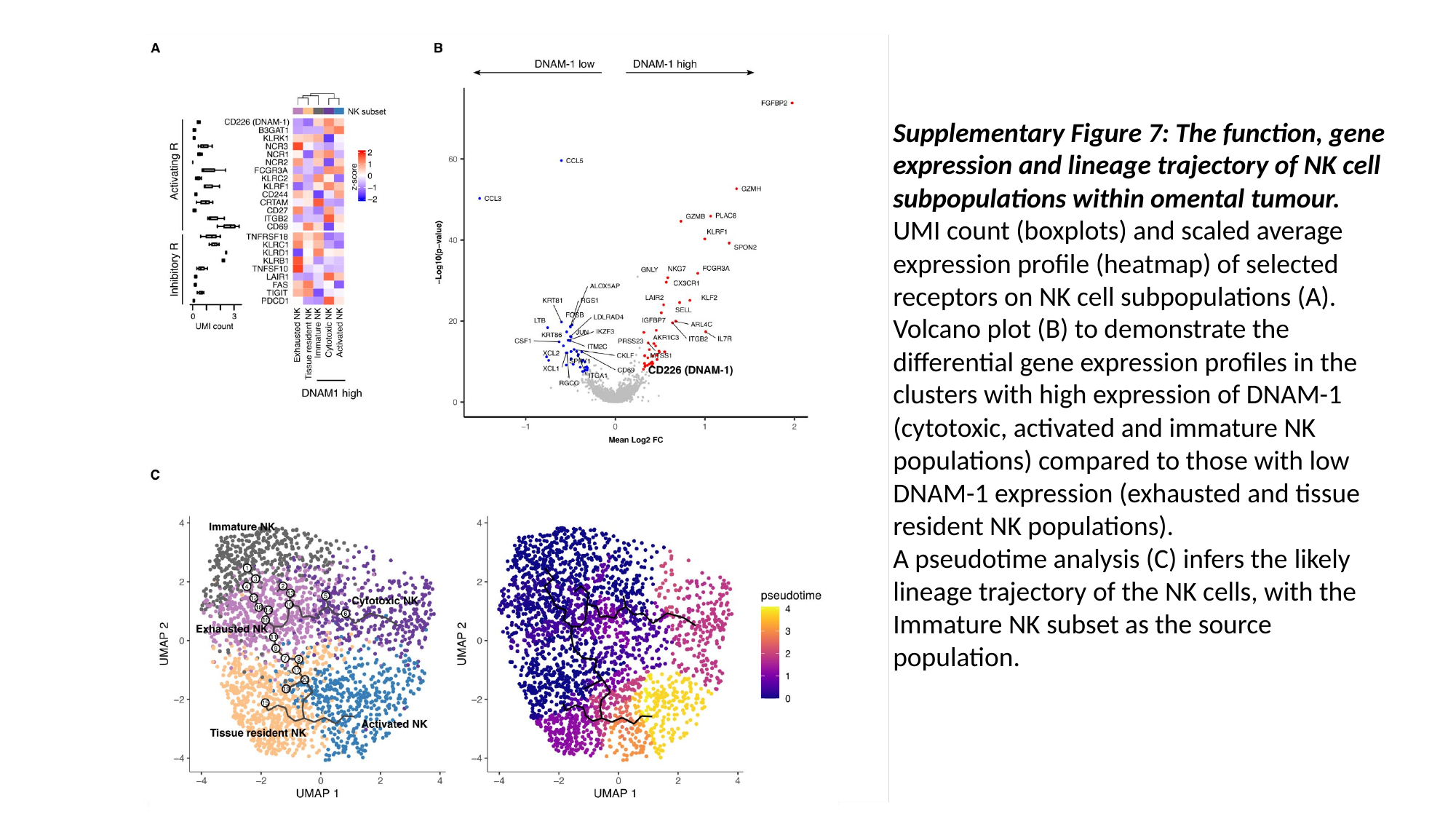

Supplementary Figure 7: The function, gene expression and lineage trajectory of NK cell subpopulations within omental tumour.
UMI count (boxplots) and scaled average expression profile (heatmap) of selected receptors on NK cell subpopulations (A).
Volcano plot (B) to demonstrate the differential gene expression profiles in the clusters with high expression of DNAM-1 (cytotoxic, activated and immature NK populations) compared to those with low DNAM-1 expression (exhausted and tissue resident NK populations).
A pseudotime analysis (C) infers the likely lineage trajectory of the NK cells, with the Immature NK subset as the source population.

## Slide 8
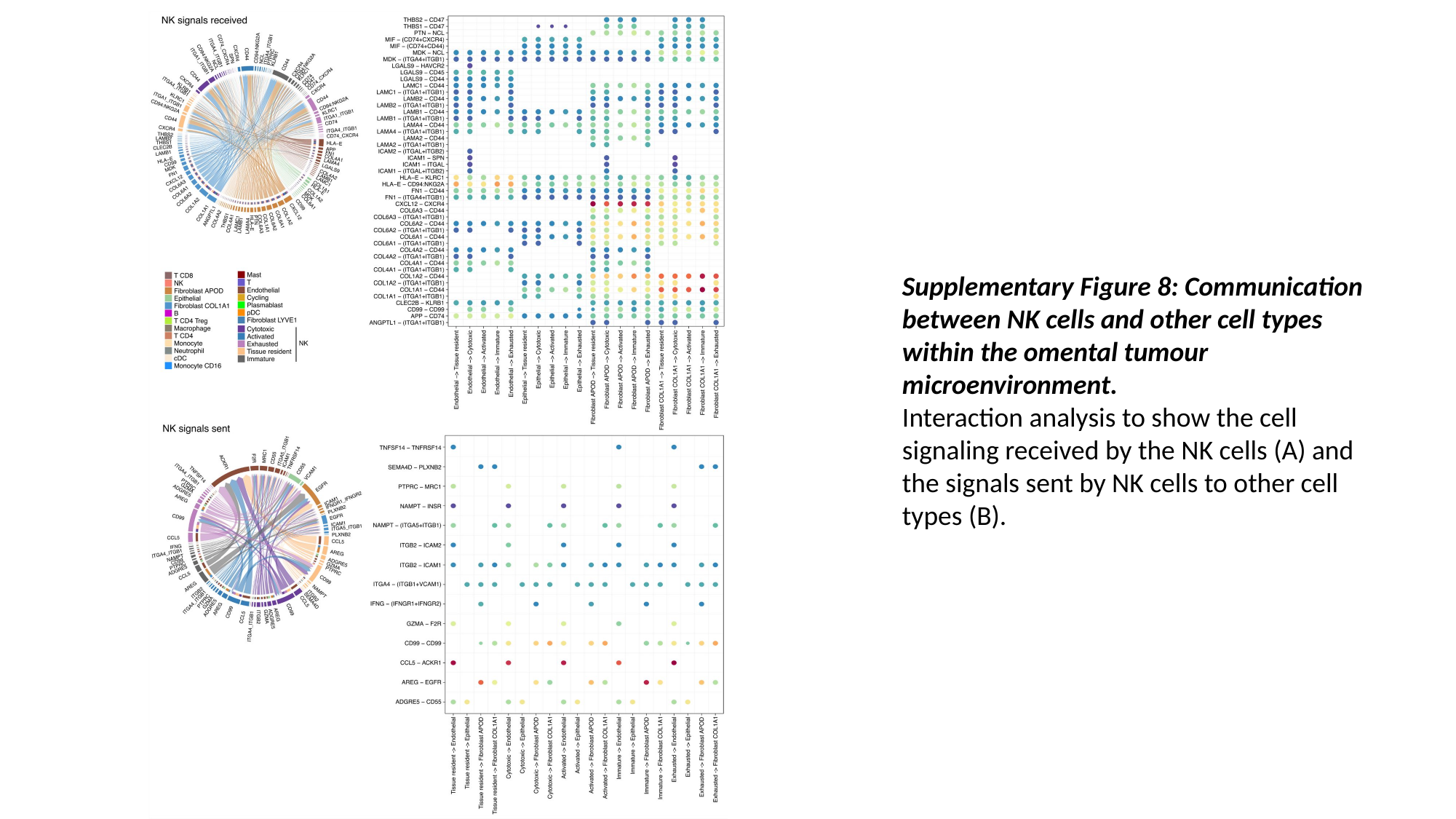

Supplementary Figure 8: Communication between NK cells and other cell types within the omental tumour microenvironment.
Interaction analysis to show the cell signaling received by the NK cells (A) and the signals sent by NK cells to other cell types (B).
